# The constitutive activation of *STAT3* gene and its mutations are at the crossroad between LGL leukemia and autoimmune disorders

**DOI:** 10.1038/s41408-024-00977-0

**Published:** 2024-01-18

**Authors:** Gianpietro Semenzato, Giulia Calabretto, Antonella Teramo, Vanessa Rebecca Gasparini, Elisa Rampazzo, Gregorio Barilà, Renato Zambello

**Affiliations:** 1 University of Padova, Department of Medicine, Hematology Unit, Padova, Italy; 2 Veneto Institute of Molecular Medicine, Padova, Italy; 3 Present Address: Hematology Unit, Ospedale S. Bortolo, Vicenza, Italy

**Keywords:** Chronic lymphocytic leukaemia, Lymphoproliferative disorders

## Abstract

Type T Large Granular Lymphocyte Leukemia (T-LGLL) is a chronic disorder characterized by the abnormal proliferation of clonal cytotoxic T cells. The intriguing association of T-LGLL with autoimmune and inflammatory diseases, the most prominent example being rheumatoid arthritis, raises questions about the underlying pathophysiologic relationships between these disorders which share several biological and clinical features, most notably neutropenia, which is considered as a clinical hallmark. Recent progress in molecular genetics has contributed to a better understanding of pathogenetic mechanisms, thus moving our knowledge in the field of LGL leukemias forward. Focusing on the constitutive activation of STAT3 pathway and the well-established role of *STAT3* mutations in T-LGLL, we herein discuss whether the T cell clones occurring in comorbid conditions are the cause or the consequence of the immune-inflammatory associated events. Overall, this review sheds light on the intricate relationships between inflammation and cancer, emphasizing the importance of the STAT3 gene and its activation in the pathophysiology of these conditions. Gaining a deeper understanding of these underlying mechanisms seeks to pave the way for the development of novel targeted therapies for patients affected by inflammation-related cancers.

## Introduction

Molecular genetics not only contributes to understanding the pathogenesis of diseases but is also becoming steadily more relevant in clinical practice. Genomic profiling enables the distinction of discrete disease subsets and provides information on the outcome of various lymphoid neoplasms [1]. Consequently, the genetic approach now dictates the classification of many hematological malignancies and plays a crucial role in their clinical management. Large granular lymphocytes leukemias (LGLL) are no exception. In fact, the discovery of STAT3 mutations has not only expanded our understanding of LGL leukemogenesis but has also improved the classification of these disorders and provided valuable insights for patient management [2].

Changes of the immune system lead to increased vulnerability to infectious diseases, cancer and autoimmune pathologies, with inflammation and neoplasia being two interconnected processes. Chronic non-resolving smoldering inflammation in particular drives malignant progression, and the tumor microenvironment is now recognized as an essential component of neoplasia. Inflammatory cells, especially macrophages, interact with tumor cells and stroma, contributing to the generation of an immunosuppressive microenvironment. A crucial question that arises is whether inflammation alone is sufficient for cancer development or if a genetic event is mandatory. Furthermore, we need to address whether inflammation favors adaptive immunity or, conversely, tumor development [3]. In other words we must strike a balance between the cancer-promoting with cancer-inhibiting aspects of inflammation responses. In this scenario, the association of T-LGLL with autoimmune and inflammatory diseases represents an intriguing model for dissecting the pathways that connect inflammation and cancer.

T-LGLL is a rare clonal disease that typically exhibits a clinically indolent course and that often develops silently. When symptomatic, it primarily manifests through neutropenia-related symptoms, requiring therapeutic intervention either initially or eventually [4]. T-LGLL is strongly associated with several accompanying conditions including immunoinflammatory and autoimmune diseases [4, 5], myelodysplastic neoplasms (MDS) [6–9], aplastic anemia (AA) [9–11], and pure red cell aplasia (PRCA) [11, 12]. A significant proportion of T-LGLL patients harbor LGL clones with somatic *STAT3* mutations [13] and those with these mutations are more likely to present with rheumatoid arthritis (RA) compared to those without *STAT3* mutations [14]. Given this strong co-occurrence, we will herein discuss the role of *STAT3* gene and its related pathway in T-LGLL, particularly in relationship to the conditions that sometimes associate with this leukemia such as autoimmune diseases. By delving deeper into this issue, we can make further progress in advancing our understanding of the intricate interconnections between inflammation and cancer.

## Signal transducer and activator of transcription 3 (STAT3)

*STAT3* is a gene whose mutations might result in different clinical presentations. The dominant negative form (Loss-of-Function, LOF) causes autosomal hyper-IgE syndrome [15], while somatic point changes (Gain-of-Function [GOF] variants) are a distinctive feature of LGLL [13], a disorder engendered by the abnormal expansion of cytotoxic lymphocytes. Additionally, the germline STAT3 LOF variant results in a multisystem disease part of primary immune regulatory disorders characterized by lymphoproliferation and polyautoimmunity [16].

**Fig. 1:**
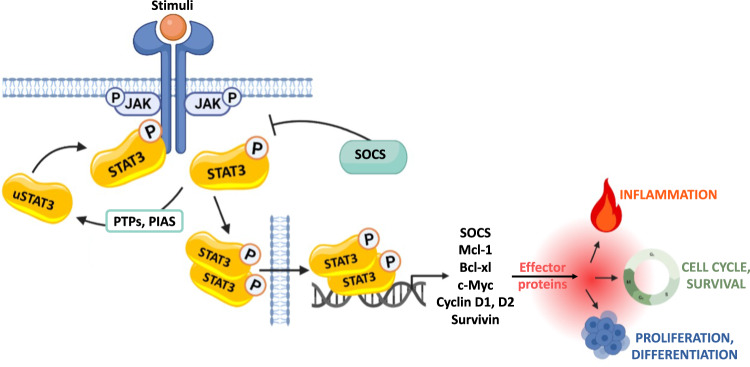
The JAK STAT signaling pathway. Activation of the receptor-associated tyrosine kinases JAKs is promoted by the binding of its ligand that induces receptor dimerization and cross-phosphorylation. Unphosphorylated STAT monomers (uSTAT3), once recruited to the phosphorylated receptors, undergo tyrosine phosphorylation and dimerize. The STATs dimers can then translocate from the cytoplasm into the nucleus, where they regulate fundamental biological processes, by activating the expression of genes involved in cell differentiation, proliferation, inflammation and apoptosis. The JAK-STAT pathway is finely regulated on multiple levels, through different mechanisms, including the suppressor of cytokine signaling (SOCS) proteins, the protein inhibitor of activated STAT (PIAS) family and protein tyrosine phosphatases (PTPs).The figure has been created by BioRender.com.

STAT3 is part of the Janus kinase (JAK)/STAT pathway which is one of the most complex transcription regulators participating in a variety of physiological processes. It mediates signal transduction from the cell membrane to the nucleus in multiple intracellular and extracellular activities. Among the 7 members of the STAT protein family, STAT3 is a cytosolic protein that, upon activation by many pro-inflammatory cytokines, hormones and growth factors, is tyrosine-phosphorylated (pY^705^STAT3) by JAK. It then dimerizes and translocates to the nucleus to activate target genes transcription, Mcl1, c-Myc, cyclins D1 and D2, Bcl-xl among others (Fig. 1). The STAT pathway is therefore involved in the regulation of crucial genes related to extremely widespread functions including cell proliferation, differentiation, survival, inflammation, immunity, and metastasis, thereby being central in many physiological and pathological processes [17].

Negative regulation of JAK/STAT pathway activation primarily depends on the suppressor of cytokine-signaling (SOCS) family members. Specifically, SOCS3 proteins are synthesized after IL-6 induction via pSTAT3 and can inhibit IL-6/STAT3 signaling in a classic feedback loop. Therefore, finely tuning STAT3 activation, SOCS3 plays a key protein in lymphocyte homeostasis, with its down expression inversely correlating with T-cell proliferation levels [18].

Reprogrammed metabolism is a hallmark of cancer and several lines of evidence indicate that STAT proteins can shape distinct metabolic processes that regulate tumor progression and therapy resistance [19]. In addition to their role in tumor cells, STATs also play a role in the accompanying inflammation, particularly in all nonmalignant cells in the cancer-associated microenvironment. These cells can drive the development and progression of tumors by affecting extracellular matrix production, angiogenesis and metabolic milieu. Intracellular STAT3 activation in immune cells also exerts suppressive effects on antitumor immunity and leads to the differentiation and mobilization of immature myeloid-derived cells and tumor-associated macrophages. Consequently, numerous studies have shown that STAT pathway is overactivated in multiple types of tumors and its targeted inhibition remains the main goal for designing new molecules that improve therapeutic strategies in the context of neoplasia [20].

## T-large granular lymphocyte leukemia

Type T Large Granular Lymphocyte Leukemia (T-LGLL) is a chronic disorder involving blood, bone marrow, spleen and liver characterized by the abnormal proliferation of clonal T cells with cytotoxic activity usually presenting with cytopenias [4, 21]. This atypical proliferation is the result of a prolonged antigenic stimulation leading to an apoptotic dysregulation mainly engendered by the constitutive activation of survival pathways, notably the JAK/STAT pathway.

**Fig. 2:**
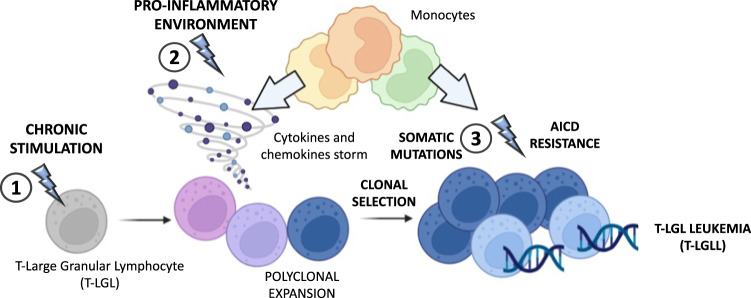
Schematic representation of T-LGLL pathogenesis. The first step of T-large granular lymphocytes (T-LGL) proliferation is related to a chronic viral or auto-antigen stimulation (1), leading to T-LGLs activation. Then a storm of pro-inflammatory cytokines and chemokines, particularly produced by monocytes, sustains and fosters T-LGL proliferation (2). A third event leads to a monoclonal expansion leading to resistance to the physiological mechanism of activation induced cell death (AICD), to dysregulation of pro- and anti-apoptotic genes expression, as well as to somatic mutations, such as STATs mutations (3). These events promote the survival and the maintenance of the neoplastic clone, leading to the establishment of T-LGLL. The figure has been created by BioRender.com.

T-LGLL is characterized by a variety of clinical presentations, ranging from indolent to aggressive, life threatening conditions and biological/molecular features are similarly divergent. T-LGLL is actually two distinctive clinicopathological subtypes, the most common CD8+ T-LGLL and the less frequent CD4+ T-LGLL, with CD4 being expressed either alone or in association with CD8^dim^. Likewise T-LGLL can be further divided by surface TCR chains expression with Tα/β- and Tγ/δ-LGLL subsets [22]. Even if the etiology of LGLL is elusive, evidence is accumulating on a putative antigen exposure that starts producing large amount of effector molecules in a context of a favorable microenvironment characterized by hyperactive signaling pathways and abnormal cytokines’ production, with the genetic landscape driving and/or sustaining chronic LGL proliferations (Fig. 2).

The disease is diagnosed in asymptomatic individuals in nearly 30% of cases during routine laboratory tests, with lymphocytosis representing the only hematological abnormality. Patients may run asymptomatic for many years; however, in 60% of cases therapy is needed during the course of disease, mostly for cytopenia-related manifestations. Only a minority of patients experience severe hematological diseases, such as fever, anemia and thrombocytopenia. In particular, severe neutropenia and anemia are found in about 20% and 10% of cases, respectively. Symptomatic patients show clinical complications closely linked to low neutrophils count and T-LGLL is frequently associated with a wide spectrum of accompanying diseases, in particular autoimmune disorders, most of them involving connective tissue [4]. In Western patients a strong association has been detected with RA [5] and some patients have been diagnosed as Felty syndrome [23] which is distinguished by splenomegaly, neutropenia and RA. Furthermore, T-LGL clones have been reported in bone marrow failures, such as MDS [6–9], AA [9–11] and PRCA [11, 12], this latter association being more commonly seen in Asians. These comorbid conditions seemingly share a common immune mediated pathogenesis as well as clinical presentations, sometimes making the differential diagnosis challenging [24]. Other less frequent immune mediated accompanying diseases include Sjogren syndrome [25] and celiac disease [26].

Under certain circumstances, an exaggerated immunodominant oligo/monoclonal expansion in response to antigenic challenge can be so prominent as to be detectable above the polyclonal background, thus mimicking a true leukemia. As a consequence, the clone’s persistence must be documented for 6 months to rule out reactive conditions. These ongoing lymphocyte expansions are referred as T-cell clone of uncertain significance (T-CUS) and represent a potential precursor of T-LGLL [27]. More precisely, T-CUS defines T-cell clones exhibiting immunophenotypic and molecular patterns closely resembling those of T-LGLL. However the affected individuals lack other laboratory or clinical features supporting a diagnosis of T-cell malignancy, particularly cytopenias, bone marrow invasion and related symptoms.

## Autoimmune disorders

Autoimmune diseases are a heterogeneous group of disorders, including clinically systemic illnesses (e.g., systemic lupus erythematosus) and organ specific illnesses (e.g., multiple sclerosis). These diseases arise from the action of multiple genetic, immunological and environmental risk factors. Functional defects of immune system explain the dampened responses to pathogens in these patients and infection represents one of the major causes of mortality. Additionally, the use of immunosuppressive drugs further increases the risk. From a pathogenetic point of view these diseases are characterized by the loss of tolerance against self-antigens. T cells (regulatory, memory, helper and effector lymphocytes) are among critical players of this process. The loss of protection against self-directed immune attack and aberrant T cell responses against autoantigens lead to tissue destruction in several organs: kidney, skin, joints among others. Antigen spread, superantigens as well as reactiveness with viral antigens have been proposed as underpinning pathogenetic events of autoimmunity but the exact triggering mechanisms that contribute the loss of tolerance and the sustained autoantibody production are still unknown [28].

Genetic predisposition to autoimmunity has been reported to involve multiple genes that regulate the activities of immune cell populations. However, autoimmunity can also result from single-gene mutations affecting key regulatory pathways. Infection seems to be a common trigger for autoimmune disease, and the microbiota can also influence pathogenesis [29].

Since the time of Rudolf Virchow in the 19th century, the links between cancer, autoimmune and inflammatory processes, and their contribution to tumor initiation and progression, have long been recognized but the reasons of this association are poorly understood [30]. The prevailing assumption to interpret this coexistence rests on the possibility that specific genes and cellular mechanisms involved in immunological self-tolerance, when disrupted by inherited or acquired mutations, cause autoimmune disease break out and further cellular mechanisms foster their maintenance. For a long time, it has been suggested that lymphoid clones, evading tolerance mechanisms, interact with specific checkpoints triggering the autoimmune process that ultimately results in the damage of self-tissues [31, 32]. Along this line of reasoning, abnormal lymphocyte clones can evade self-tolerance both by affecting one of the multiple stages occurring in the evolution of immune responses including the effector B and T cell functions (inhibition of B cell receptor, of Treg cells and of PD-1 among others), as well as the control of antibody extravasation and inflammation in tissues. Blocking these checkpoint mechanisms might prevent the clearance of self-antigens from the body and thus induce autoimmune conditions.

As mentioned earlier, RA is the most frequent autoimmune disorder associated to T-LGLL, being reported in approximately 25% of patients across the different case series [33]. Clonal CD8 expansions, resembling T-LGLL but not meeting the diagnostic criteria, have been reported in RA [34]. These clones should be interpreted as T-CUS, which are likely to be a consequence of the chronic auto-stimulation occurring in the disease.

Evidence of a genetic link between the two diseases comes from the observation that Human Leukocyte Antigen (HLA)-DR4 has been detected in 90% of patients with concurrent T-LGLL and RA, while T-LGLL without RA express HLA-DR4 in a percentage similar to the general population (30%) [35]. Furthermore, a similar serological pattern of autoantibodies has been reported between T-LGLL and RA [23]. There is no a defined timing in the appearance of both disorders; T-LGLL might precede the clinical manifestation of RA or might be diagnosed several years after the diagnosis of RA. Sometimes the two disorders are detected simultaneously [4, 35].

## STAT3 constitutive activation and somatic mutations in disease states

A constitutive activation of the STAT3 signaling pathway has long been demonstrated in T-LGLL [36, 37] and the discovery of *STAT* mutations [13] has been instrumental in expanding our understanding of LGL leukemogenesis but also in improving the classification of these disorders [1, 2]. Although they are not pathognomonic for T-LGLL and can be detected in various hematological and non-hematological malignancies [38], *STAT3* mutations are a useful molecular marker for T-LGLL management in the appropriate clinical context [1, 39]. Mutations on *STAT3*, mostly located within the SRC homology (SH2) domain, as well as in other regions of the protein, but more rarely, have been detected in approximately 50% of T-LGLL and are recognized as the commonest GOF genetic lesions identified to date in these patients [13, 40–45]. These mutations, which enhance STAT3 activation through stabilization of protein dimerization, are now regarded as the biological hallmark of the disease thereby providing valuable information for the management of LGLL patients.

The assessment of mutational landscape is now recommended for accurate characterization of LGLL patients [39]. In fact, a strong correlation has been established between the presence of *STAT3* mutations and neutropenia, which is the clinical hallmark of the disease [13, 41, 42, 44]. Among a series of 101 patients tested, 89% of those with *STAT3* mutations were neutropenic, with a highly statistically significant *p* value [41]. Based on this observation, the impact of mutations has been evaluated on the overall survival and the presence of *STAT3* mutations has been found to correlate with a worse outcome [45]. T-LGLL patients with *STAT3* mutations are more likely to develop RA than those without mutations [14, 42]. The incidence of genetic lesions in T-LGLL varies widely across different cases series reported in the literature, and this variability is consequent to the different methods used, ranging from Sanger/targeted amplicon sequencing to whole exome/genome sequencing [22]. Although the frequency range is wide, it is interesting to note that the presence of *STAT3* and *STAT5B* mutations is almost mutually exclusive [22].

*STAT3* mutations have not been detected in T-CUS but only a very limited number of case series are available [24] and we would not be surprising to find such mutations in this precursor state. In fact, the presence of genetic abnormalities in other premalignant conditions, such as Monoclonal B cell Lymphocytosis (MBL) and Monoclonal Gammopathy of Undetermined Significance (MGUS), similar to those observed in the overt diseases (i.e., Chronic Lymphocytic Leukemia and Multiple Myeloma) support the hypothesis that preneoplastic conditions possibly represent the cognate malignancy in their infancy [46]. Currently, the diagnosis of overt malignancy in all these premalignant states is based on clinical features rather than biological aberrancies. The discovery of discrete markers could improve the diagnostic cut-offs to identify patients who are likely to progress to the overt malignant counterpart and those who are not, especially in individuals who are up on the tightrope.

As mentioned earlier, not all LGLL harbor *STAT3* mutations, and not all T-LGLL cases associated with other diseases have *STAT3* mutations [40–45], which rises the issue of the specificity of these genetic alterations. Since the STAT pathway is constitutively activated in all T-LGLL cases, it can be inferred that other events are responsible for this activation. In fact, *STAT5B* mutations have been particularly demonstrated in the T-CD4 subset of the disease [43, 47]. Additionally, other less frequent point mutations, insertions, or deletions have been detected, along with other recurrently mutated genes, including *TNFAIP3* and less frequently *BCL11B*, *FLT3* and *PTPN23* in T-LGLL [48]. These additional mutations could represent the missing link to explain why patients without *STAT3* mutations show a hyperactive STAT3 pathway.

Furthermore, both the overexpression of miR-181a [49], which results in hyperactive STAT3 and ERK1/2 pathways, and an activated IL-6-STAT3 loop [37] provide additional explanations for the hyper-activation in *STAT3* non-mutated patients. According to the above interpretation, the fact that immunosuppressive therapy is unable to reduce the level of mutated clone points towards the search of novel targets [50].

## The “chicken and egg conundrum”

The issue of clonality and its relationship with cancer is a matter of debate and is indeed mostly intricate to address when relying on mutations. Unlike germline mutations that are inherited, somatic mutations accumulate in healthy subjects during life, especially in normal aging as well as in a wide variety of benign inflammatory conditions currently acquired. Most of them, particularly when detected at low frequencies, are of little or no functional consequences [51].

Genetic abnormalities, and in particular *STAT3* GOF mutations, which keep lymphocyte activation of T cell clones out of check, have been regarded as one of the putative explanations for the development of autoimmunity. These expanding cytotoxic T cell clones perpetuate the tissue damage both directly and by inducing autoantigens through a process of protein citrullination [52]. In line with this hypothesis, in a mice model with germline *STAT3* GOF mutations, Masle-Farquhar et al. demonstrated that the resulting effector CD8 + T cell clonal accumulation contributes to autoimmune pathology [53]. Furthermore, a T cell clone-mediated platelet activation and apoptosis with an antibody-independent mechanism of platelet destruction has been reported in patients with immune thrombocytopenia characterized by T-cell clones persisting over many years [54]. Interestingly, these T CD8+ clones were more prominent in patients with refractory disease and expanded when the platelet count was low.

**Fig. 3:**
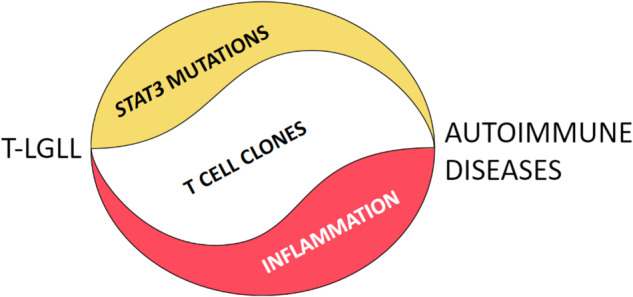
The chicken and egg conundrum.

However, since preclinical studies suggest that inflammation themselves induces clonal expansions [55, 56], the question arises of whether mutations might be the consequence of an abnormal induced immune event rather than the cause, as previously suggested, of the accompanying autoimmune disorders. In fact, the signals and self-reactive clonal/oligoclonal cytotoxic T lymphocyte (CTL) driven expansions generated during immune response might not disappear when the inflammation is cleared, thus overriding the relentless proliferation of lymphocytes. This can then lead to mutations that confer an advantage to clone development and persistence, ultimately unleashing the emergence of a lymphoid malignancy (Fig. 3).

The earlier mentioned timing of appearance of associated disorders (previous, concurrent or subsequent) does not help to solve the matter [4, 35]. Furthermore, whether abnormal LGL clones are sufficient to induce autoimmune pathology is a preliminary question that remains unanswered. To that end, targeted STAT3 small-molecule inhibitors should be evaluated in patients with autoimmune disorders to check the effect of removal of the rogue clonal population.

A reasonable hypothesis rests on the possibility that *STAT3* mutation testing can help distinguish between T cell clones causing the associated disease from those that are its consequence. Although data are still limited, up to now *STAT3* mutations have not been detected in T-CUS and patients with *STAT3* mutations develop RA and neutropenia more often than patients without these mutations [13, 57, 58]. Thus, in general, a more clinically symptomatic disease is likely to be associated to the presence of *STAT3* mutations and the evidence that neutropenia is more detectable in more severely affected patients suggests that *STAT3* aberrancies might be responsible. Further studies on appropriate series of cases are needed to substantiate this hypothesis.

The alternative hypothesis cannot be excluded that we are dealing with a vicious cycle in which self-perpetuating events induce clinical problems, particularly neutropenia.

## STAT3 and the pathogenesis of neutropenia

It is worth mentioning that the gene mutations, *STAT3* and *STAT5B* among others, in T-LGLL are unlikely to be the initial inciting trigger of leukemic process (Fig. 2). Instead, they are rather believed to represent an acquired event during the disease that fosters clone development [59]. Furthermore, these mutations are associated with several biological activities. Among these, the STAT3-miR146b-FasL axis has been shown to be paramount in the pathogenesis of neutropenia which is also a common feature to all other diseases accompanying/overlapping T-LGLL. Specifically, neutropenic T-LGLL patients exhibit large amounts of plasmatic soluble FasL leading to Fas-mediated apoptosis of neutrophils [60, 61]. It has been demonstrated that increased FasL production is a result of Human antigen R (HuR) starvation. HuR is an essential FasL mRNA stabilizer and target gene of a noncoding RNA (miR-146b) which in neutropenic patients is down-regulated due to STAT3-dependent miR-146b promoter hypermethylation [62].

The Fas/FasL pathway, being involved in the activation-induced cell dead (AICD) mechanism, plays a central role in inducing cytopenia. However, the pathogenesis of LGLL-related neutropenia has been reported to be multifactorial, including immune alterations, bone marrow infiltration/substitution, splenic sequestration and cell-mediated cytotoxicity [63].

Consistent with the detection of neutropenia in LGLL patients, *STAT3* mutations also appear to be associated with worse clinical course in patients with autoimmune disorders, particularly in RA and Felty syndrome [13]. These mutations also contribute to early-onset multiorgan autoimmunity including neutropenia [64]. This finding suggests that these conditions overlap to a certain extent, likely representing a continuum of a clinicopathologic process with a common pathogenesis, leading to immune-mediated neutrophil destruction. Data in MDS and immune mediated AA are likely to be similar but up to now clinical correlations are still scarce. The STAT3 signaling, activated by the *STAT3* SH2 domain, is a recurrent event also in the lymphocytic variant-Hyper Eosinophilic Syndrome [65, 66]. This disorder is characterized by a clonal proliferation of T cells that produce Th2 cytokines, thereby stimulating a paraneoplastic proliferation of non-neoplastic eosinophils and is often associated with severe infections.

Given that genetic and epigenetic alterations regulate the entire immune cell repertoire, influencing immunocompetent cells and their anti-tumor immune responses, it is essential to further specify the cross-talk between leukemic cells and nonleukemic mononuclear cells in microenvironment. This can be accomplished by capitalizing on the unprecedented improvement of high throughput sequencing and multi-omics single-cell analyses. Such approaches allow for the study of various cell populations including Th17 cells and monocytes, among others, which contribute to the production of pro-inflammatory molecules that are central in triggering clonal LGL expansion and its persistence [67, 68].

Another robust pathogenetic link between LGLL and autoimmune diseases lies in the role of Interleukin-15 (IL-15) in both conditions. IL-15 is a proinflammatory cytokine that plays a central role in the pathogenesis of both LGLL [69, 70] as well as of several autoimmune diseases [71]. Belonging to the IL-2 family of cytokines IL-15 promotes activation of T cells, NK-cells, neutrophils, macrophages, and dendritic cells. Notably IL-15 is involved in the development of autoimmune disease by enhancing the activation and maintenance of IL-17 producing T cells [71]. Further evidence of the dependency on IL-15 of T-LGL leukemic cells comes from a recent phase I/II clinical trial demonstrating that treatment with BNZ-1, a novel multi-cytokine inhibitor blocking the common gamma chain receptor of IL-2, IL-9 and IL-15, shows therapeutic effects in these patients [72].

## Therapeutic implications

Understanding how the relationships between leukemia and inflammation impact on anti-cancer immune response may provide insights into orchestrating more effective immune strategies. In fact, unlike most lymphoproliferative disorders, indication for treatment is not the disease bulk but is rather focused on concurrent cytopenias.

Currently, no specific therapies are available for these disorders. T-LGL leukemia is a rare disease, and lack of large prospective trials has prevented the establishment of a standard therapy. Moreover, the limited understanding of the disease’s etiopathogenesis hinders the design of accurate treatment targets, leaving patients with limited options for therapy. The most common conventional treatments rely on immunosuppressors, either as monotherapy or as a part of a multitherapy regimen which includes corticosteroids methotrexate, cyclosporine, and cyclophosphamide. However, these immunosuppressors yield unsatisfactory clinical results with response rates ranging from 55% to 80%, the purpose of treatments being to correct cytopenia and reduce the proliferation of clonality, rather than eliminate the rogue clones. In addition, they are often poorly tolerated and may be inadequate in patients with severe cytopenias. Taking all these factors into consideration, the need for more effective and potentially personalized treatments remains unmet in these patients.

New therapeutic approaches of these borderline and overlapping disorders should build upon recent achievements in terms of molecular pathogenesis. STAT proteins are able to shape distinct metabolic processes that regulate tumor progression by transducing signals from cytokines, growth factors and their receptors. They also regulate genetic programs involved in immune cells at the transcriptional and epigenetic level. In this latter regard, since epigenetic modulation relies on reversible interactions with different structures, it is also modifiable by environmental influences, including drugs. As a consequence, DNA and RNA methylation-demethylation, and chronic remodeling by histone acetylation-deacetylation represent a promising approach to prevent tumor progression [73].

**Fig. 4:**
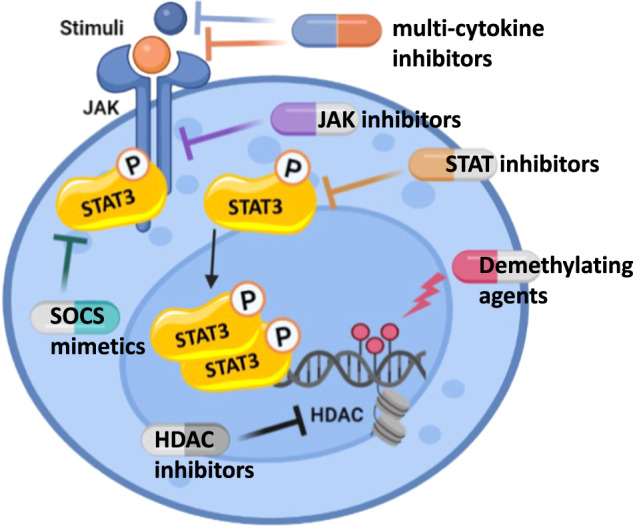
New therapeutic approaches. Current attempts are based on recent advances in molecular pathogenesis, particularly those that involve the pathways that connect inflammation and cancer (see text). The figure has been created by BioRender.com.

Given their central role in promoting cancer cell survival, invasion, therapy resistance and immunosuppression via multiple mechanisms, molecules that target proteins of the STAT family are being utilized to develop novel modulators of the pathway, including JAK inhibitors, STAT inhibitors and SOCS mimetics [74, 75], as well as histone deacetylase inhibitors [76]. Additionally, attempts may be made to exploit T-cell-receptor constant-domain immunotherapy [77]. These molecules hold the potential to become alternative therapeutic options for innovative treatments of these disorders [78]. The STAT signaling pathway, in particular, has been considered as a potential target for therapeutic intervention. Promising results have been reported with the use of JAK inhibitor Ruxolitinib in patients with refractory T-LGLL [79] as well as in those with lymphocytic variant-Hyper Eosinophilic Syndrome associated with clonal T cell proliferation [80]. Of course, a more comprehensive understanding of distinct molecular mechanisms of JAKs pathways and related mutations might prompt new insights to the LGLL community and inspire the development of new drugs with greater specificity, tailored for subgroups of T-LGLL that must be precisely identified in advance [22] (Fig. 4).

Further approaches to enhance our therapeutic arsenal rely on a comprehensive evaluation of associated mutational profiles using high-depth next-generation sequencing (NGS). The ultimate goal is to identify different T-LGL patients who may exhibit distinct responses to treatments. Additionally, the analysis of the STAT variant allele fraction (VAF) percentage could be helpful in assessing tumor burden potentially identifying patients who would benefit most from specific therapies as well as providing insights into the response to treatment.
